# Mental Health Nurses’ and Allied Health Professionals’ Individual Research Capacity and Organizational Research Culture: A Comparative Study

**DOI:** 10.1177/23779608241250207

**Published:** 2024-05-13

**Authors:** Geoffrey L. Dickens, Maria Avantaggiato-Quinn, Sara-Jaye Long, Mariyana Schoultz, Nicola Clibbens

**Affiliations:** 1 Department of Nursing Midwifery and Health, 5995 Northumbria University, Newcastle Upon Tyne, UK; 2 5633 Cumbria Northumberland Tyne & Wear NHS Trust, Newcastle Upon Tyne, UK

**Keywords:** Research culture, research capacity, mental health, mental health services, mental health nurses, evidence-based practice

## Abstract

**Introduction:**

Healthcare professionals have development needs related to their consumption, use, and practice of clinical research. Little is known about these issues in mental health services specifically.

**Objectives:**

A survey of healthcare staff working in an NHS Mental Health and Disability Trust in England was conducted to describe research capacity and culture compared with previously reported samples, and to examine subgroup differences.

**Methods:**

An online questionnaire was utilized. The main measure was the Research Capacity and Culture tool comprising measures of individual's perceived research skills and of team and organizational research culture. Previous studies using the same measure were systematically identified, and pooled results, weighted by sample size, were calculated. Analyses were descriptive (current sample versus previous results) and inferential (comparisons between demographic and professional groups within the current sample).

**Results:**

*N* = 293 people completed the survey. The median item scores were poorer than those of pooled samples from studies reporting median item scores on 39/51 (76.5%) occasions and poorer than those pooled samples of studies reporting mean item scores on 51/51 (100.0%) occasions. Individual capability for research was in the ‘less than adequate’ range more than in previous samples (71.4% vs. 42.9%). For team culture items, the proportions were 84.2% vs. 78.9%, while most responses about organizational culture were in the ‘adequate’ range (55.6% vs. 66.7%). Staff >20 years employment had poorer perceptions of team and organizational culture.

**Conclusion:**

Perceptions of individual research capacity and team and organizational culture were poor compared with previous studies, most of which were conducted in non-mental health settings. There is need for development of research capacity and culture in mental health services including opportunities to develop basic research skills through to strategic developments to promote clinical academic careers. There is considerable room for improvement in the way organizations support research and signpost opportunities.

## Introduction

Incorporating new knowledge from high quality research is key to evidence-based healthcare (Lehane et al., 2019). Research can extend knowledge about intervention content and effectiveness, illness prevention, diagnosis, and patient experience; in turn, these can facilitate healthier, longer lives, better experiences of health services, and improved use of valuable resources (Jordan et al., 2019). Engagement of health services in research activity, specifically in intervention trials, is significantly associated with indicators of mortality and care quality (Jonker & Fisher, 2018). Research using other paradigms including collaborative and action research also contributes to improved patient outcomes and greater uptake of evidence-based care processes (Boaz et al., 2015). The number of studies running in health services, as opposed to the number of participants recruited, is positively associated with better results from staff and patient surveys about information provision, teamwork, confidence in doctors, and inpatient experience (Jonker et al., 2019). A recent qualitative systematic review (Newington et al., 2021) found that research activity – as opposed to research findings – impacted positively on patients, service provision, economics, and staff recruitment and retention. In addition, the best available research evidence should inform the preparation of healthcare professionals in educational curricula content (Lehane et al., 2019), and should be available to practitioners who have the skills to identify, evaluate and implement it (Guyatt et al., 2000). At more advanced levels, healthcare practitioners are expected to participate in and lead on the identification of evidence gaps, the design and planning of research to meet those gaps, securing funding, conducting studies, analyzing data, and disseminating research results (NHS, 2017). In the UK, strategies to enhance the involvement of nurses (NHS England and NHS Improvement, 2021) and allied health professionals (NHS Health Education England, 2022) have been recently launched.

Given the above context, it is important to equip the clinical workforce with research-related knowledge, skills, and opportunities appropriate to their role, and to effectively utilize the capabilities of those with existing advanced research-related training. Practitioners of some professions have traditionally been viewed as less research active in general, and less active in research leadership specifically (Wenke & Mickan, 2016). For example, while non-medical healthcare professionals are striving to increase their role in research leadership and are targeting clinical academic roles in England to rise to 1% of all roles by 2030 (Westwood et al., 2018), 5% of UK medical consultants already work in such roles (Healthcareers NHS, 2017; National Institute for Health Research, 2016).

## Review of Literature

Reflecting this ambition, recent studies have sought to establish research capacity and capability among non-medical healthcare professionals including allied health professionals, nurses and midwives, their teams and employing organizations (see Table 1). Some have used the empirically validated Research Capacity and Capability Questionnaire (RCC; Holden et al., 2012a). Table 1 summarizes 37 papers identified in a systematic literature search for primary research using the RCC for the current study. Twenty-seven studies were conducted in Australia, eight in the UK, and two in the US. Most involved Allied Health Professionals (AHPs) and most participants were AHPs. Only one sample, the smallest of those included, was drawn solely from a mental health service (Migliorini et al., 2022) while two further studies included <10% individuals working in mental health services within their samples (Comer et al., 2022; Gill et al., 2019). Mental health professionals, and mental health nurses in particular, are understudied.

**Table 1.** Previous Published Studies Using the RCC and Reporting Median (IQR) or *M*(SD) Scores per Item.

Study	Country	Sample	*N* Samples	Response rate	Setting	Result reported
Alison et al. (2017)	Australia	*N = *276 allied health professionals	1	54%	Local Health District	Median IQR I T O item
Becker et al. (2017)	US	*N = *73 trainee and qualified medical staff	1	30%	Integrated health care system	Median IQR I item
Borkowski et al. (2017)	Australia	*N = *136 allied health professionals	1	46%	Regional health service	Mean SD I T O item/total
Boyd et al. (2019)	US	*N = *24 registered dietitian nutritionists	1	6.4%	National survey	Median I T O Item
Brandenburg et al. (2021)	Australia	*N = *225 medical doctors	1	10.1%	Statewide publicly funded tertiary health service	Median IQR I T O item
Chinn et al. (2023)	UK	*N = *278 healthcare staff	1	5.5%	Scottish NHS Health Board	Median IQR I T O Item
Comer et al. (2022)	UK	*N = *3276 allied health professionals (*n = *306 working in mental health trusts)	1	-	National survey	Median IQR I T O item
Cordrey et al. (2022)	UK	*N = *93 allied health professionals/support workers	1	33.4%	NHS Foundation Trust	Median IQR I T O item
Crombie et al. (2021)	Australia	*N = *216 allied health professionals across 2 iterations	2	-	Regional health service	Median IQR I T O item
Elphinston & Pager (2015)	Australia	*N = *60 psychologists	1	26.1%	Large metropolitan public health setting	Mean SD I T O totals only.
Frakking et al. (2021)	Australia	*N = *75 healthcare staff	1	47.6%	Secondary 265-bed hospital	Median I T O item
Gill et al. (2019)	Australia	*N = *776 healthcare workers	1	5.3%	Region including 8 health service partners, academic health science center and network	Median I T O item
Gimeno et al. (2021)	UK	*N = *92 allied health professionals	1	55%	Tertiary children's hospital	Median I T O item
Harper et al. (2022)	Australia	*N = *62 occupational therapist across two iterations	2	59%/49.1%	Tertiary hospital	Mean I T O item
Holden et al. (2012a)	Australia	*N = *69 allied health professionals. Control and intervention groups x2 iterations each	4	-	Staff working in teams in one geographical district	Mean I T O item/ total
Holden et al. (2012b)	Australia	*N = *134 healthcare workers	1	-	One Local Health District	Median I T O item
Howard et al. (2013)	Australia	*N = *130 nutritionists and dieticians	1	40%	State-wide survey	Mean I O item/total
Johnson et al. (2022)	Australia	*N = *278 pharmacists	1	43.4%	State-wide survey	Mean (SD) I T O total.
Lazzarini et al. (2013)	Australia	*N = *70 podiatrists across 2 iterations	2	64%/55%	State-wide survey	Median I T O item
Lee et al. (2020)	Australia	*N = *393 ahps, nurses, medics reported separately	3	7%	Local health district	Median I T O item
Lieschke et al. (2022)	Australia	*N = *816 nurses and midwives	1	10%	One Local Health District	Mean I O total
Luckson et al. (2018)	UK	*N = *224 nurses and allied health professionals in two hospitals (reported separately)	2	24%	Two hospitals	Mean (SD) I T O total
Matus et al. (2019)	Australia	*N = *302 allied health professionals	1	30%	One health service in one state	Median I T O item
Matus et al. (2021)	Australia	*N = *320 allied health professionals	1	-	One health service (5 hospitals plus community) in one state	Median I T O item
Migliorini et al. (2022)	Australia	*N = *59 social workers and occupational therapists	1	22%	Area-based Public Mental Health Service	Median I T O item
Raschke et al. (2022)	Australia	*N = *1243 research-eligible employees in two locations (results reported separately)	2	-	Local Health District	Mean (SD) I T O item/ total
Rathi et al. (2023)	Australia	*N = *191 allied health professionals	1	12%	National private health service	Mean (SD) I T O item/ total
Ray et al. (2022)	UK	*N = *62 respiratory nurses	1	-	National survey	Mean I T O item
Wenke et al. (2018)	Australia	*N = *16 allied health professionals across two iterations	2	-	One health service	Mean (SD) I item
Williams & Lazzarini (2015)	Australia	*N = *232 podiatrists	1	6%	National survey	Median I T O item
Williams et al. (2015)	Australia	*N = *520 allied health professionals	1	-	Statewide	Median I T O item
**Excluded studies**						
**Study**	**Country**	**Sample**	***N* Samples**	**Response rate**	**Setting**	**Reason for exclusion**
Goncalves & White Personal Communication	UK	*N = *531 healthcare staff	1		Hospitals, universities, ambulance service, conference	Not peer reviewed. Exclude
McEvoy et al. (2023)	Australia	*N = *112 people with lived experience as mental health consumers	1	-	Purposive and snowball sampling from lived experience networks	RCC extensively modified. Exclude.
Pain et al. (2018)	Australia	*N = *482 allied health professionals	2	43%/37%	Regional tertiary hospital	RCC modified to 5-points. Exclude.
Palmer et al. (2023)	UK	*N = *416 healthcare staff	1	8.6%	NMAHP professionals and students at a university and an acute healthcare organization	RCC rescaled to 9 points. Exclude.
Pighills et al. (2013)	Australia	*N = *86 occupational therapists	1	57%	Statewide survey	RCC rescaled to 5-points. Exclude
Raschke (2017)	Australia	*N = *691 healthcare staff	1	-	Rural Local Health District	RCC rescaled to 9-points. Exclude

I – RCC Individual factor; T – RCC Team factor; O – RCC Organizational factor; item – scores per item presented; total – scores presented as mean per item total score per factor.

Given the current lack of information, and in a policy context in which non-medical professionals are encouraged to develop research skills (NHS England and NHS Improvement, 2021; Royal College of Occupational Therapists, 2019), the current paper describes a survey of research capability and capacity conducted in a UK NHS Mental Health and Learning Disability Trust. The overall aim was to explore perceptions of individual research capacity, team and organizational culture among all non-medical health professionals. The term ‘Nursing, Allied Health Practitioners Plus’ (NAHP+) is used to describe the target sample since it extends beyond legally defined categories of ‘nurse’ and ‘allied health professional’ to encompass groups including psychologists, social workers, and pharmacists. A complete list of participating professionals can be seen in Table 2. The study was conducted to aid planning for increased future research capacity and impact. Objectives were: i) describe participants self-reported capacity to contribute to research activity, compare this between demographic and professional groups, and with results from previous research; ii) describe participants perceptions of team and organizational research culture making similar between group and across study comparisons; and iii) describe participants’ reports about their personal research interests, their perception of opportunities to get involved in research, and of individual and organizational barriers to, and motivators of, research activity.

**Table 2.** Respondent Characteristics, *N* = 293.

Professional group	*n* (%)
*Registered nurses*	
Registered nurse	114 (38.9)
Total nursing	114 (38.9)
*Allied health professionals*	
Occupational therapist	58 (19.8)
Speech and language therapist	20 (6.8)
Physiotherapist	14 (4.8)
Art therapist	9 (3.1)
Dietitian	5 (1.7)
Total allied health	106 (36.2)
*Psychology*	
Psychologist	37 (12.6)
Assistant psychologist	2 (0.7)
Psychological therapist	2 (0.7)
Psychotherapist	2 (0.7)
Total psychology	43 (14.6)
*Other*	
Pharmacist	18 (6.1)
Social worker	8 (2.7)
Employment specialist	2 (0.7)
Counsellor	1 (0.3)
Mental health practitioner	1 (0.3)
Total other	30 (10.2)
Years qualified	
0–2	18 (6.1)
3–5	31 (10.6)
6–10	57 (19.5)
11–20	95 (32.4)
21+	86 (29.3)
Missing	6 (2.0)
Highest qualification	
Up to diploma	26 (8.9)
Bachelors	153 (52.2)
Masters	72 (24.9)
Doctoral	37 (12.6)
Missing	5 (1.7)
Pay Band	
3–4	2 (0.7)
5–6	106 (36.1)
7	94 (32.1)
8a/8b	65 (22.2)
8c/8d	20 (6.9)
Missing	6 (2.0)
Research is part of role	
Yes	128 (43.7)
No	162 (55.3)
Missing	3 (1.0)
Proportion of role doing research (%)	
0	143 (48.8)
1–10	114 (38.9)
11–20	21 (7.2)
21–30	4 (1.4)
>30	9 (3.0)
Missing	2 (0.7)
Research discussed in appraisal	
Yes	64 (21.8)
Only if I raise it	128 (43.7)
No	97 (33.1)
Missing	4 (1.4)
Self-assessed research level	M = 2.4 *SD *= 1.2
0	18 (6.1)
1	55 (18.8)
2	89 (30.4)
3	72 (24.6)
4	48 (16.4)
5	8 (2.7)
Missing	3 (1.0)
Currently studying	
Yes	27 (9.2)
No	261 (89.1)
Missing	5 (1.7)

## Methods

### Design

A cross-sectional survey design with quantitative (objectives 1 and 2) and qualitative (objective 3) elements was used. Comparison data from previous literature was obtained from a literature search that followed principles outlined in the Preferred Reporting Items for Systematic Reviews and Meta Analyses. The study report complies with the Consensus-based Checklist for Reporting Of Survey Studies (CROSS; Sharma et al., 2021).

### Sample

The study was conducted in Cumbria Northumberland Tyne & Wear NHS Mental Health Trust (CNTW), a large mental health and disability service provider in North East England serving a population of 1.7 million. CNTW employs 8,000 staff of whom around 4,000 met inclusion criteria for participation. A sample size calculation revealed that *n = *351 participants would produce results with a margin of error of 5% with a 95% confidence level. The sample was a self-selecting convenience sample. Information about the survey was posted regularly over 12-weeks in Spring 2022 on the Trust's bulletin email and intranet dashboard. A hyperlink was provided to the survey on Microsoft Office 365. Respondents were invited to provide an email address to participate in a prize draw; these were decoupled from survey data prior to analysis to preserve anonymity.

### Inclusion/Exclusion Criteria

Eligible participants were Trust employees (any age) who met the following criteria: nurses registered with the Nursing and Midwifery Council, or any practitioner of the 14 allied health professions identified by NHS England (n.d.) and regulated in the UK by either the Health and Care Professions Council, or the General Osteopathic Council; or psychologists, social workers, pharmacists, or other degree level non-medical practitioners including assistant psychologists and counsellors. A full list of participating professions is presented in Table 2.

***Ethical considerations.*** The study was a service evaluation and did not require ethical approval. It was registered and approved via CNTW's Service Evaluation Approval system (SER-20-095 on 19/3/21). All study elements were conducted in accordance with the Declaration of Helsinki. Full study information was provided, consent was implied by completion of the study questionnaire. Participants, who were anonymous, could stop participating at any time.

***Measures.*** The Research Capacity and Culture questionnaire (RCC) was the main measure used (Holden et al., 2012a). It has been found to have strong internal reliability (Cronbach's α= 0.95–0.96) and test-retest reliability ICC = 0.77–0.83) for all three of its factors. Each of the 51-items is scored from 1 to 10. Items relate to i) individual research skills and success (‘individual factor’; 14 items); ii) team level research skills and success (‘team factor’; 19 items); and iii) organization level research skills and success (‘organizational factor’; 18 items). Respondents can answer ‘unsure’; such responses are reported as frequencies and do not contribute to the calculation of average item scores. Items about registering audit or service evaluation and gaining Trust support for research were added. The single-item Skills, Capability, and Organizational Research Readiness’ (SCORR) scale (Iles-Smith et al., 2019) was also administered. The outcome is measured on a 6-point criterion referenced scale from 0 (requires support to gain knowledge from evidence on practice/research and apply it to practice) to 5 (leads the generation of knowledge through research activity, leads and develops clinical research, obtains research funding etc). To answer objective 3, participants were asked to describe their own areas of research interest, their awareness of research involvement opportunities available in the Trust, and their experience of barriers to, and motivators of, research activity individually, at team and organizational level; response to these items was binary (yes/no) but each encouraged additional free-text responses. These data were complementary to quantitative data. Demographic and professional information, information about current and previous engagement with and experience of research activity were also gathered (see Table 2).

***Benchmark data.*** To facilitate comparisons between the current sample and previous reports a search of electronic literature databases (CINAHL, AMED, PsycArticles and MEDLINE) was conducted using the terms “RCC Tool” and “Research Capacity and Culture Tool” in conjunction with the name of the tool's author (“Holden”; example search included in supplementary material). Forward and backward citation searching was conducted of papers that reported studies using the RCC or literature reviews including reports of use of the RCC. The results from six studies (total *N = *2318) were excluded because the researchers had amended the response scale of the RCC making direct comparison and synthesis with other results problematic. Hence, 31 previous studies (see Table S1) reporting RCC results for 42 samples (*N *= 10920) were identified (some studies presented results for two iterations or multiple groups of staff); results for 23 samples (*N *= 7634) were reported as the median (IQR) at individual item level, those for 15 samples (*N *= 1908) as item level mean scores, and those for four samples (*n *= 1378) as factor level mean per item scores. No studies contributed to both median and mean benchmark scores.

### Statistical Analysis

***Benchmark scores.*** Data were entered into three Microsoft Excel spreadsheets (*Median* [*IQR*] per item scores, *M* [*SD*] item scores, and *M*[*SD*] factor level scores) along with information about *n* responses per item once ‘unsure’ responses were accounted for). Item level data were synthesized by calculating *Median* [*IQR*] and *M* [SD] scores per item weighted by *n* responses. *Median Median* scores (i.e., the middle item score when all weighted item scores were placed in order of magnitude) were calculated at factor level. Total factor level weighted *M*[*SD*] per item scores were calculated by combining results from studies with item level reporting and those with factor level only reporting weighted by total study sample size since factor level only scores had insufficient information about ‘unsure’ responses. Resulting weighted measures (possible range 1–10) were categorized thus: < 4 ‘less than adequate’, 4–6.99 ‘adequate’, and >6.99 ‘more than adequate’ (Holden et al., 2012a).

***Quantitative survey data.*** Data were entered into IBM SPSS statistics software (Version 25.0). To describe the sample and examine research culture, descriptive statistics (frequencies, proportion, *M* (*SD*) were calculated. Missing RCC data were managed as follows: *Median (IQR)* for each item was calculated based on available data excluding ‘unsure’ responses and missing responses. Factor per-item *M(SD)* scores were calculated based on responses of participants who had a maximum number of missing responses as follows: individual factor two items, team and organizational factor three items. Comparisons of *M(SD)* per item score between groups derived from demographic/professional characteristics were made using parametric tests (independent samples t-tests and One Way ANOVAs). Statistical significance was set at *P *< .05. Comparisons between current sample item *M (SD)* and *Median (IQR)* item and factor level scores with weighted benchmark scores were descriptive (frequencies and proportions).

***Qualitative data.*** Qualitative data were analyzed on a per item basis. An inductive/deductive two-step content analysis method was used. First, half of the responses were independently subjected to inductive open coding. Results were compared, discussed, and codes organized into clusters. Second, the remaining responses were deductively coded, where possible, into the clusters. Where they could not be coded into existing clusters they were discussed, and a consensus decision was made about how to code them or whether the existing coding structure needed to be amended. Rigor and trustworthiness of the analysis process (Creswell & Path, 2017) was ensured through independent coding by two researchers, team discussion of the final coding scheme, and consideration of how many respondents contributed free text responses in each area, and for each cluster.

## Results

### Sample Characteristics

*There* were 299 survey responses; four did not meet profession-related inclusion criteria and two contained no completed RCC items leaving *N *= 293 for analysis (response rate 7.3% based on estimated population size of 4000). There was 6.4% missing RCC data. Factor *M(SD)* and Median (IQR) per-item scores were calculated based on the responses of 291 (individual), 276 (Team) and 270 (Organizational) participants. Based on Trust data, nurses were underrepresented in the final sample (response rate 4.4%) while allied health professionals were over-represented (23.0%). Based on analysis of organizational factor data, nurses and allied health nurses were equally likely to meet the threshold for inclusion (15 items completed) indicating no non-response bias. Sample characteristics are presented in Table 2. The most common research-related activities in which respondents affirmed current engagement were ‘using research evidence to inform my clinical practice’ (81.2%) ‘involvement in clinical audit or service evaluation’ (28.7%), and ‘I raise awareness of clinical trials to patients in my area’ (23.5%). The least commonly engaged with activities were taking on principal or chief research investigator roles (both <4.0%). The most common past 12-month self-reported research activity was ‘None’ (30.4%). Engagement in service evaluation, quality improvement, evidence-based practice implementation and clinical audit was affirmed by 25.2–29.0% of respondents. A minority of respondents had disseminated research via conference presentation (8.2%) or authorship/co-authorship of research papers (10.9%).

Professional groups were equally likely to hold Masters’ qualifications (Χ^2 ^= 0.19, df = 3, *P *= 0.98); registered nurses and allied health professionals were equally likely to hold doctorates (Χ^2 ^= 0.99, df = 1, *P *= 0.32). Numbers of respondents prepared to doctoral level in the other groups were too low to include in this analysis. Professional groups were similar on self-assessed SCORR research level (F(2,289) = 1.37, df = 2, *P *= 0.26), years qualified (Fisher Exact Test *P = *0.70), highest qualification (*Χ^2 ^*= 3.08, df = 3, *P = *0.38), current pay band (*Χ^2 ^*= 2.77, df = 3, *P = *0.43), in having research as a part of their role (*Χ^2 ^*= 1.03, df = 3, *P = *0.80), current student status (Fisher Exact Test, *P = *0.78), and whether research formed a regular part of their annual appraisal (Fisher Exact Test *P = *0.40).

Of 36 respondents educated to doctoral level, 17 (47.2%) said research accounted for no part of their role; 14 (38.9%) said it accounted for 1–10% of their workload. Similarly, of those prepared to masters’ level, 88.9% reported <10% of their role involved research. Sizeable proportions of those educated to masters’ (25.4%) or doctoral level (38.9%) said research was not discussed in their annual appraisal; even more in each group (40.8% and 47.2% respectively) said research was only discussed if they raised the issue.

### Research Objectives

***Objective 1: individual research capacity***. *M*(*SD*) per-item RCC individual factor score was 3.8 (1.9) compared with 5.0 (2.4) from benchmark studies and 10/14 items had mean scores categorized as less than adequate (see Table 3) compared with just one from the benchmark studies. The weighted *Median* scores from previous studies were classed less than adequate (6/14), adequate (6/14), and more than adequate (2/14); overall *Median (IQR)* per item score was 2.5 (1–5.5) compared with 4 (2–6) from previous studies where the *Median* score was reported. In direct comparisons, *Medians* from the current sample were poorer than those from previous research for 9/14 items and *M* poorer for all 14 items. The highest rated individual items in the current sample were: ‘finding relevant literature’ and ‘critically reviewing the literature’ (*Median *= 6). The lowest rated item was ‘securing research funding’ (*Median *= 1). Ranked order of items between the current and previous studies were significantly correlated (Spearman's *rho *= .911, *P *< .001). Two additional non-RCC items achieved scores of *Median *= 2: ‘knowledge of how to register an audit’ and ‘registering a service evaluation’, while ‘gaining organizational support for research’ achieved *Median = *1.

**Table 3.** RCC Individual Factor Results.

Previous research^a^		RCC internal reliability at individual level α=.95 I have skills related to…	*Current study*		
Weighted Median (IQR)	Weighted Mean		Median (IQR)	Mean (SD)	Unsure *n* (%)
7 (5–8)	7.0 (1.7)	1. Finding relevant literature	6 (5–8)	6.1 (2.1)	4 (1.4)
7 (5–8)	6.5 (1.7)	2. Critically reviewing the literature	6 (4–7)	5.6 (2.2)	3 (1.0)
5 (2–7)	5.3 (2.4)	3. Using a computer referencing system (e.g., Endnote)	3 (1–6)	3.9 (2.7)	6 (2.0)
3 (2–6)	4.6 (2.3)	4. Writing a research protocol	2 (1–5)	3.2 (2.4)	5 (1.7)
1 (1–3)	3.2 (2.0)	5. Securing research funding	1 (1–2)	2.0 (1.8)	8 (2.7)
2 (1–5)	4.2 (2.4)	6. Submitting an ethics application	2 (1–4)	2.7 (2.3)	6 (2.0)
4 (2–7)	5.1 (2.2)	7. Designing questionnaires	4.5 (2–6)	4.5 (2.4)	7 (2.4)
5 (3–7	6.0 (2.2)	8. Collecting data e.g., surveys, interviews	5 (3–7)	5.0 (2.6)	5 (1.7)
3 (1–6)	4.8 (2.4)	9. Using computer data management systems	2 (1–5)	3.2 (2.5)	6 (2.0)
4 (2–6)	4.6 (2.3)	10. Analyzing qualitative research data	3 (1–6)	3.9 (2.5)	4 (1.4)
4 (2–6)	5.0 (2.4)	11. Analyzing quantitative research data	2 (1–4)	2.7 (2.0)	17 (5.8)
4 (2–7)	5.0 (2.4)	12. Writing a research report	3 (1–6)	3.8 (2.7)	6 (2.0)
3 (2–5)	4.2 (2.4)	13. Writing for publication in peer-reviewed journals	2 (1–5)	3.1 (2.6)	4 (1.4)
2 (1–5)	4.0 (2.3)	14. Providing advice to less experienced researchers	2 (1–5)	2.9 (2.5)	5 (1.7)
4 (2–6)	5.0 (2.4)	Individual factor per item	2.5 (1–5.5)	3.8 (1.9)	

*Note*. Weighted mean item scores based on studies where individual item mean scores reported (mean *n = *1688 per item); weighted medians based on mean *n = *7000 responses per item; weighted mean total score based on *n = *2603 responses.

^a^ Weighted means and medians derived from previous studies (see Table 1).

The sole between group difference on individual factor mean-per-item score was that those who rated themselves the most research-skilled (SCORR scores 4 and 5) scored *lower* than those who reported themselves least skilled (levels 0 and 1) (see Table 4).

**Table 4.** RCC Factor Mean-per-Item Scores by Demographic and Professional Group.

	Individual		Team		Organization	
Professional Group	Mean (SD)	Test	Mean (SD)	Test	Mean (SD)	Test
Nursing (115)	3.77 (1.82)	F (3,291) = 1.19 P = .15	3.50 (2.13)	F (3,273) = 0.15 P = .93	4.21 (2.31)	F (3,267) = 0.94 P = .42
Allied (136)	3.54 (1.94)		3.33 (2.23)		3.95 (2.31)	
Psychology (43)	3.98 (1.98)		3.54 (1.95)		4.35 (2.24)	
Other	4.12 (1.62)		3.43 (1.94)		4.74 (2.43)	
Years qualified						
0–5 (49)	3.75 (1.87)	F (3,282) = 1.47 P = .22	3.81 (2.13)	F (3,258) = 4.66 P = .003^a^	4.67 (2.41)	F (3,252) = 4.20 P = .006^b^
6–10 (56)	4.19 (2.10)		3.73 (2.32)		4.23 (2.41)	
11–20 (95)	3.76 (1.77)		3.33 (2.08)		4.08 (2.23)	
21+	3.51 (1.85)		2.01 (1.15)		2.69 (1.34)	
Highest qualification						
Diploma (26)	4.38 (2.10)	F (3,283) = 1.66 P = .18	3.89 (1.97)	F (3,268) = 0.85 P = .47	4.40 (2.21)	F (3,262) = 1 P = .33.15
Bachelors (152)	3.61 (1.69)		3.39 (2.12)		4.22 (2.30)	
Masters (72)	3.67 (1.89)		3.22 (2.09)		3.80 (2.25)	
Doctoral (37)	4.07 (2.32)		3.75 (2.25)		4.63 (2.53)	
Current pay band						
3–5 (27)	3.88 (1.94)	F (3,282) – 1.02 P = .38	3.33 (2.14)	F (3,268) = 1.06 P = .37	4.43 (2.48)	F (3,261) = 0.82 P = .49
6 (81)	4.02 (2.01)		3.77 (2.15)		4.47 (2.42)	
7 (94)	3.70 (1.75)		3.36 (2.07)		4.09 (2.26)	
8+ (84)	3.52 (1.85)		3.18 (2.14)		3.93 (2.33)	
Research part of role						
Yes (128)	3.95 (1.94)	t (287) = 1.55 P = .12	3.48 (1.96)	t (272) = 0.30 P = .77	4.26 (2.30)	t (267) = 0.44 P = .66
No (161)	3.61 (1.80)		3.41 (2.12)		4.13 (2.34)	
Research discussed in appraisal						
Yes (64)	3.43 (1.75)	F (1,287) = 12.41 P=,12	3.00 (1.91)	F (2,270) = 2.62 P = .07	3.70 (2.27)	F (2,264) = 2.35 P = .10
No (96)	3.87 (1.82)		3.77 (2.13)		4.52 (2.34)	
Only if I raise it (128)	3.74 (1.90)		3.32 (2.10)		4.10 (2.25)	
Self-assessed research level						
0–1 (73)	4.15 (1.94)	F (2,286) = 3.38 P = 0.04	3.44 (2.28)	F (2,271) = 0.03 P = .97	4.13 (2.50)	F (2,265) = 0.03 P = .97
2–3 (162)	3.67 (1.88)		3.44 (2.09)		4.20 (2.27)	
4–5(58)	3.32 (1.47)		3.36 (1.98)		4.22 (2.22)	
Currently studying						
Yes (27)	4.24 (2.21)	t (289) = 0.201 P = .14	3.68 (2.40)	t (270) = 0.55 P = .58	4.53 (2.62)	t (265) = 0.73 P = .47
No (284)	3.68 (1.82)		3.43 (2.10)		4.17 (2.29)	

^a^ Post hoc Tukey HSD indicates significantly lower score for those in group Years Qualified 21 + than all other categories.

^b^ Post hoc Tukey HSD indicates significantly lower score for those in group Years Qualified 21 + than all other categories.

***Objective 2: team and organizational research culture.*** *M(SD)* per-item RCC team factor score was 3.5(2.1), and 16/19 items were categorized as less than adequate compared with *M (SD) *= 4.8 (2.7) and 2/19 weighted *M(SD)* scores rated inadequate in previous studies (see Table 5). *Median (IQR)* team factor score in the current sample was 2.5 (1–5) and 16/19 items were rated less than adequate on this measure compared with 3(1–6) and 10/19 in benchmark studies. *Medians* from the current sample were poorer than those from previous research for 14/19 items and *M* poorer for all 19 items. The highest rated *Median* team factor items in our sample were: ‘has team leaders that support research’, ‘undertakes planning that is guided by evidence’, and ‘supports a multi-disciplinary approach to research’ (*Median *= 4); the lowest rated item was ‘has applied for external funding for research’ (*Median *= 1). Items were ranked similarly in the current study and in previous studies (Spearman's *rho *= .73 (*P *< .001). The only group difference on ratings that those with 21 + years since qualification were less positive than three groups with less time since qualification (see Table 4).

**Table 5.** RCC Team Factor Results.

Previous research^a^		RCC internal reliability at team level α=.98	Current study		
Weighted Median (IQR)	Weighted Mean (SD)		Mdn (IQR)	Mean (SD)	Unsure *n* (%)
3 (1–6)	4.2 (2.2)	1. My team has adequate resources to support staff research training	3 (1–5)	3.5 (2.3)	11 (3.8)
2 (1–4)	3.7 (2.0)	2. My team has funds/equipment/admin to support research activities	2 (1–4)	2.9 (2.0)	13 (4.4)
3(1–5)	4.3 (2.2)	3. My team participates in team level planning for research development	2 (1–5)	3.0 (2.2)	15 (5.1)
3 (1–5)	4.7 (2.3)	4. My team ensures staff involvement in developing that plan	3 (1–5)	3.3 (2.4)	15 (5.1)
5 (2–7)	6.2 (2.5)	5. My team has team leaders that support research	4 (2–7)	4.6 (2.9)	12 (4.1)
4 (2–6)	5.3 (2.4)	6. My team provides opportunities to get involved in research	3 (1–5)	3.6 (2.6)	13 (4.4)
5 (3–7)	5.9 (2.4)	7. My team undertakes planning that is guided by evidence	4 (2–6)	4.2 (2.6)	13 (4.4)
3 (1–5)	4.4 (2.3)	8. My team has consumer involvement in research activities/planning	2 (1–5)	3.0 (2.2)	16 (5.5)
2 (1–6)	4.6 (2.5)	9. My team has applied for external funding for research	1 (1–3)	2.5 (2.4)	13 (4.4)
5 (2–7)	5.6 (2.6)	10. My team conducts research activities relevant to practice	3 (1–6)	3.9 (2.9)	17 (5.8)
4 (1.75–7)	5.5 (2.4)	11. My team supports applications for research scholarships/ degrees	2 (1–5)	3.3 (2.8)	19 (6.5)
3 (1–5)	4.5 (2.2)	12. My team has mechanisms to monitor research quality	2 (1–5)	3.1 (2.5)	18 (6.1)
4 (1–7)	5.2 (2.5)	13. My team has identified experts accessible for research advice	2 (1–6)	3.4 (2.7)	18 (6.1)
5 (1–7)	5.3 (2.4)	14. My team disseminates research results at research forums/seminars	3 (1–5)	3.6 (2.8)	16 (5.5)
5 (2–7)	5.9 (2.4)	15. My team supports a multi-disciplinary approach to research	4 (2–7)	4.4 (2.8)	23 (7.8)
3 (1–5)	4.2 (2.3)	16. My team has incentives & support for mentoring activities	2 (1–5)	2.9 (2.3)	21 (7.2)
3 (1–7)	5.0 (2.5)	17. My team has external partners (e.g., universities) engaged in research	3 (1–6)	3.6 (2.8)	18 (6.1)
5 (2–7)	5.2 (2.5)	18. My team supports peer-reviewed publication of research	3 (1–6)	4.0 (2.7)	26 (8.9)
2 (1–5)	3.8 (2.4)	19. My team has software available to support research activities	2 (1–4)	2.7 (2.4)	21 (7.2)
3 (1–6)	4.8 (2.7)	Team factor per item	2.5 (1–5)	3.5 (2.1)	

*Note*. Weighted mean item scores based on studies where individual item mean scores reported (mean *n = *1438 per item); weighted medians based on mean *n = *6077 responses per item; weighted mean total score based on *n = *1636 responses.

^a^ Weighted means and medians derived from previous studies (see Table 1).

For the organizational factor, *M(SD)* total score was 4.2 (2.3), and 6/18 items were categorized as less than adequate with the remainder rated adequate (see Table 6). In contrast, the weighted *M(SD)* from previous studies was 5.4 (2.4) and no items were rated less than adequate. *Median [IQR]* in our sample was 4 (1.5–6) compared with 5 (3–7.5) in previous samples; 8/18 and 1/18 items were ranked less than adequate in respective groups. *Medians* from the current sample were poorer than those from previous research for 16/18 items and *M* poorer for all 18/18 items. In our sample, the item ‘has consumers involved in research’ (*Median *= 6) was highest rated. The items ‘has adequate resources to support staff research training’; ‘has funds/equipment/admin to support research activities’, ‘ensures staff career pathways are available in research’, ‘accesses external funding for research’, ‘has software programs for analyzing research data’, ‘has mechanisms to monitor research quality’, ‘supports applications for research scholarships/ degrees’ were rated lowest (*Median *= 3). Ranking of items in the current and previous studies were similar (Spearman's *rho *= .68, *P *= 0.02). Those with 21 + years since qualification scored significantly lower on the RCC (see Table 4).

**Table 6.** RCC Organizational Factor Results.

Previous research^a^		RCC internal reliability at organization level α=0.98	Current study		Unsure (%)
Weighted Median (IQR)	Weighted Mean (SD)		Median (IQR)	Mean (SD)	
4 (3–6)	4.8 (2.1)	1. The Trust has adequate resources to support staff research training	3 (2–6)	3.8 (2.5)	15 (5.7)
4 (2–5.5)	4.4 (2.0)	2. The Trust has funds/equipment/admin to support research activities	3 (1–5)	3.4 (2.4)	18 (6.7)
5 (4–7)	4.9 (2.2)	3. The Trust has a plan or policy for research development	4 (2–6)	4.3 (2.7)	18 (6.7)
5 (3–8)	6.3 (2.3)	4. The Trust has senior managers that support research	4 (2–7)	4.6 (2.8)	16 (6.0)
4 (2–5)	5.0 (2.1)	5. The Trust ensures staff career pathways are available in research	3 (1–5)	3.5 (2.5)	18 (6.7)
5 (3–7)	5.8 (2.3)	6. The Trust ensures organization planning is guided by evidence	4 (2–7)	4.5 (2.7)	19 (7.0)
4 (2–7)	5.4 (2.2)	7. The Trust has consumers involved in research	5 (2–7)	4.6 (2.7)	19 (7.0)
5 (2–7)	5.1 (2.2)	8. The Trust accesses external funding for research	3 (1–6)	4.0 (2.7)	18 (6.7)
7 (5–8)	6.9 (2.2)	9. The Trust promotes clinical practice based on evidence	6 (4–8)	5.7 (2.6)	18 (6.7)
6 (4–8)	6.0 (2.2)	10. The Trust encourages research activities relevant to practice	5 (2–7)	4.5 (2.8)	18 (6.7)
3 (2–6)	4.3 (2.2)	11. The Trust has software programs for analyzing research data	3 (1–5)	3.4 (2.5)	21 (7.2)
4 (2–6)	4.6 (2.3)	12. The Trust has mechanisms to monitor research quality	3 (1–5)	3.8 (2.6)	25 (8.5)
5 (3–7)	5.5 (2.4)	13. The Trust has identified experts accessible for research advice	4 (1–6)	4.2 (2.8)	25 (8.5)
5 (3–7)	6.0 (2.4)	14. The Trust supports a multi-disciplinary approach to research	3.5 (2–6)	4.2 (2.8)	19 (7.0)
5 (3–7)	5.0 (2.4)	15. The Trust has regular forums/bulletins to present research findings	4 (2–7)	4.6 (2.8)	20 (6.8)
5.5 (3–8)	5.6 (2.4)	16. The Trust engages external partners (e.g., universities) in research	5 (1–7)	4.5 (2.9)	22 (7.5)
5 (3–7)	5.8 (2.5)	17. The Trust supports applications for research scholarships/ degrees	3 (1–6)	3.9 (2.7)	19 (7.0)
5 (3–7)	5.9 (2.3)	18. The Trust supports the peer-reviewed publication of research	4 (1–6)	4.3 (2.9)	26 (8.9)
5 (3–7.5)	5.4 (2.0)	Organization factor per item	4 (1.5–6)	4.2 (2.3)	

*Note*. Weighted mean item scores based on studies where individual item mean scores reported (mean *n = *1696 per item); weighted medians based on mean *n = *5765 responses per item; weighted mean total score based on *n = *2491 responses.

^a^ Weighted means and medians derived from previous studies (see Table 1).

***Objective 3: Individual research interests, awareness of research opportunities, and barriers to and motivators for conducting research.*** Most (82.9%) respondents provided a free text response (*M* [range] length 10 words [1–103]) about their area of interest in research should they have the opportunity for involvement. Examination of coded clusters revealed common areas of interest (all >10 mentions) related to clinical audit, service improvement or service evaluation, specific diagnostic groups or services for the same (e.g., dementia, depression, psychosis), specific non-medical therapies (e.g., cognitive behavioral therapy, art therapy, neurological therapy), specific methodological approaches (clinical trials, qualitative research, participatory research, collaborative approaches), and assessment (e.g., risk assessment, suicide risk, formulation).

When asked which research-related information and opportunities were currently available in the Trust, the most recognized item (93.9%) was ‘Library services’ followed by ‘Information about what research is happening in the Trust’ (45.7%). The least affirmed were ‘Administrative support for research activities’ (7.5%) and ‘Opportunities to become a principal investigator’ (8.9%). Recognition of the ten remaining items was between 18.7% (‘Opportunities to be involved in delivering research’) and 30.7% (‘Funding within the Trust to support research’). Fifty-nine (20.1%) people provided a response to a follow-up free response item ‘Any additional comments about opportunities within the Trust?’ The most common cluster of comments related to the range of opportunities that are potentially available; these ranged from partial awareness (“*I am aware of some of these”*) to acknowledgment with caveats (“*I do not think all these opportunities are open to all staff”; “If it is not part of your formal job role but has ended up something that you have done within the service for a number of years, then nothing around the support and training elements of research listed above, get offered”*) to incongruence between the support on offer and the perception of what is actually required or desired (*“I feel that there is no dedicated time to be involved in research, without this there are no real opportunities to be involved within the trust”*; *“An essential pre-requisite is desk space and dedicated time allocated”; “Have asked for possible Masters / PhD as part of job/ time away from current role to do research and opportunities but told they do not have funding/ no interest in me doing this at this time*). There were also positive responses (*“Librarians are fantastic for supporting with literature reviews / systematic reviews”* and aspirational comments “*I would be interested in training in research from the trust and informal research supervision. I'll be keen to follow up on this”*).

Responding to stimuli describing potential barriers to research participation, participants most commonly identified ‘pressure of clinical work’ and the ‘identification of research activity as a luxury’ (57.0%), ‘desire for a work/life balance’ (42.7%), ‘not knowing where to start’ (41.6%), ‘lack of skills’ (36.5%), and ‘lack of confidence’ (36.2%). These issues were reflected in free text responses made by 76.8% of respondents to a follow-up item about their experience of the biggest barriers to research activity. Time (“*Carving out time for research activity alongside clinical and training commitments - it always seems to be bottom of the list*”; “*Clinical work prioritized always not just in a pandemic with no space given for research”*) was commonly cited as an issue. However, multiple respondents reported the lack of a research conducive culture including “*a lack of role models”*, “*failure to prioritize research”*, and “*attitude - that it is seen as a luxury. That commissioners don't value this – ‘surely that's what the university is there for - not the NHS’- type attitude”*.

Responding to items about motivators for research involvement, the most commonly affirmed were ‘to benefit service users/carers’ (80.2%); ‘to develop skills’ (78.8%), ‘increased job satisfaction’ (58.0%); and ‘problem identified that needs changing’ (47.1%). Free text responses made by 73.0% of respondents provided a similar range of views including learning and development (“*I have a desire to do it, to look at what we are doing, why we are doing it and what the outcomes are. To see if there is a better way*”), personal ambition (”*My personal ambition has helped a lot and I'm enthusiastic about encouraging and supporting others to get involved in research if they want to*”), and improving care (“*Improving outcomes for patients, showing that innovations can improve practice and outcomes*”).

## Discussion

The current paper describes a survey of NMAHP + individual research capacity and organizational research culture in a large mental health and disability NHS Trust. Results were compared with those from more than 10,000 health care employees across 42 samples in 31 previous studies. Previous studies have largely reported median per item scores while some have reported mean per item scores and a small number have reported mean per item factor scores; this has facilitated three sets of comparisons between the current sample's respondents and those from previous research. Results from all three sets of comparisons were consistent. Across the 51-items of the RCC, scores were poorer in the current sample compared with studies reporting medians in 39 cases and better in two. Compared with studies reporting means they were poorer in all 51 cases. This was reflected in factor level median and mean scores which were all poorer in comparison to previously reported results. While the reason for this clear discrepancy is not certain, it is notable that the sample examined in the current study is one of only two to be drawn entirely from a mental health organization.

In the current sample, capability in identifying and critically reviewing relevant literature were rated as adequate both (*Median *= 6); this might be expected when narratives about evidence-based practice permeate healthcare deeply (Lehane et al., 2019) and evidence-based practice narratives strongly feature in pre-registration healthcare education standards (Nursing & Midwifery Council, 2018; Royal College of Occupational Therapists, 2019). Nevertheless, in comparison studies, both items were rated as ‘more than adequate’ (*Median *= 7) indicating a poorer position in this sample.

Individual research skills items with median ratings of ‘adequate’ were about collecting data and designing questionnaires, activities which do not exclusively occur within the research domain. Other skills which are transferable between research and activities such as clinical audit or service improvement, for example data analysis, were rated as inadequate yet achieved levels in the adequate range in previous samples. Coupled with very low median ratings in the current sample related to knowledge of audit and service evaluation this is of concern. There is a need for ongoing programs of education for these groups of clinical staff that encompass evidence-based practice skills. Importantly, any such education program should seek to improve skills that are transferable between audit, service improvement and research. This may be best achieved by providing examples that are directly linked to practitioners’ own practice setting and congruent with their experience (Lyon et al., 2011).

Research skills related to writing for publication, advising others on research and securing funding were rated as less than adequate both in the current sample and previous samples. While there is no ‘correct’ proportion of employees who should have skills in these areas, this finding reflects the very low proportion of nurses and allied health professionals who have completed doctoral level studies. In the US, the number of graduations from nursing doctoral programs has remained between 611 and 804 for the decade 2012–2021(AACN, 2022). In the UK the number of PhD-prepared nurses in a typical university hospital is between 0 and 10 (Carrick-Sen, 2019). Leading on from this, a particularly notable finding was that over half of those respondents already prepared to doctoral level said that research played no part in their professional role whatever; for even more, research was not discussed, or only discussed if raised by themselves, during annual appraisals. Reflecting findings from a UK qualitative study of doctoral level nurses, it seems that their skills are under-utilized, especially when they remain in roles close to clinical practice (Hampshaw et al., 2022)

Nursing and allied health professionals have very different routes into clinical academic roles than their medical colleagues: the former group usually working for many years in clinical practice prior to developing a joint role, and the latter undertaking longer joint training from the outset of their career (Trusson et al., 2021). These are systemic issues that require addressing by professional bodies and at national policy level. Advanced practice masters’ programmes have had significant investment in England and are underpinned by four pillars: clinical practice, leadership and management, education, and research (NHS England, 2017). Findings from the current study suggest that while the advanced practice pillars imply a quarter of advanced practice relates to research, like findings from studies in non- mental health settings (Hampshaw et al., 2022; Palmer et al., 2023), this is not reflected in the day-to-day work While investment in funded pathways to clinical academic careers provides structure for joint clinical academic careers for non-medical professionals they are highly competitive, “hard to come by” (Palmer et al., 2023: p.6), and require significant preparation by applicants (NIHR & NHS Health Education England, n.d.). A requirement for employers to provide increased time and support to prepare for such opportunities is a consistent finding of studies seeking to evaluate nursing or allied health professional research capacity development (Bramley et al., 2018; Hampshaw et al. 2022; Palmer et al. 2023; Spring et al. 2023) if individuals are to apply successfully to these pathways. Research fellowship opportunities are not new in nursing and allied health professions, but recent studies identify the need for more sustained career development (e.g., Palmer et al. 2023). Importantly, successful innovative fellowships have included elements of transformational learning including mentorship and leadership (Palmer et al., 2023) alongside more traditional research training and opportunities to lead specific projects (Spring et al. 2023).

Organizational research culture was rated as adequate for more than half of the items in the current sample, just two fewer than weighted medians from previous samples. Still, the results suggest that this NHS Trust has considerable room to develop how it supports research culture for non-medical staff and, importantly, how information about research is communicated. A need for improved collaborations between university and health services leaders has been identified, to not only drive the development of joint clinical academic roles but to ensure that they are sustained over time through secure organizational ‘buy in’ (Spring et al. 2023) as the research culture takes time to change (Bramley et al., 2018; Palmer et al. 2023).

Team and organizational culture was rated similarly by profession, pay band, qualification level, and self-assessed research level; however, those with most experience (>20 years) held poorer views of both than their less experienced colleagues. This group might have been less exposed to evidence-based practice narratives during professional training. Disillusionment or failure to keep abreast of developments may also be a factor. There is an equal need to engage with longstanding employees around research ability and culture as there is to provide support for those earlier in their professional career. Those with longer professional experience are likely to be clinically expert and possess transferable knowledge and skills for research that could be optimized through research training and mentorship (NIHR, 2022).

Some of the above issues have been recognized by key research funders in England through ringfenced funding for underrepresented professions including nursing and allied health professionals (National Institute for Health and Care Research, 2023a,b). Such funding is important to address this study's finding, and those in comparator studies, that nurses and allied health professionals lack skills and confidence in applying for research funding. Whilst similarity between groups suggests that a united approach to developing nursing and AHP research careers could be viable, rather than diluting and replicating efforts, as recognized by the National Institute for Health and Care Research (2022), there is a need to ensure the distinct voice of each profession is acknowledged by having separate funding calls for nurses to allied health professionals.

A key question arising from the study is: does the derivation of our sample solely from a mental health and learning disabilities service provider explain dissimilarities with previous findings? Compared with Migliorini et al.'s (2022) study, conducted in a mental health service in Australia, median ratings were marginally less poor on individual skills (9/14 inadequate medians), but clearly better on team (12/19 inadequate medians) and organizational culture (3/18 inadequate medians). Compared with the current sample, Comer et al.'s (2022) UK-based study comprising approximately 10% of its sample from mental health services had fewer median ratings of inadequate on individual skills (6/14) and a similar number of inadequate medians on the remaining two scales (24/37). Clearly the current study cannot pinpoint a causal role for the mental health setting but it remains an issue for consideration and future comparative research.

While the results discussed this far are based on the quantitative element of the study, respondents were also provided with the opportunity to volunteer free text information about their own research interests, their perception of research opportunities within the Trust and of barriers to and motivators for research involvement. This facilitated some potentially rich complementary data to inform planning for future strategic research developments. Regarding the barriers to and motivators for conducting research, many participants identified insufficient time to be involved in research since priority was given to clinical activities. This resonated with the findings that research was not usually part of appraisal discussion. A key finding of a similar study in an acute care setting identified the need for research to be better integrated into clinical roles (Palmer et al., 2023). Staff shortages and lack of access to research funding were also linked by respondents to a need to prioritize clinical workload. This suggests that research in the organization is not seen as priority and this aligns with previous findings in UK and worldwide literature (Harrison, 2005; Hutchison & Johnston, 2006, Kanmounye et al., 2020). Since research skills are key to the development of evidence-based practice, a dialog is needed between staff, service mangers and academics to encourage incorporation of research activities as part of the routine clinical work in order to support the organization and the staff to meet their objectives about evidence-based practice.

For many clinicians, the infrastructure to facilitate research is inaccessible due to insufficient support or a lack of clear pathways to develop research skills (Oulton et al., 2022). Staff feel stymied by prevailing attitudes that research is solely an academic concern. This requires challenging with narratives about how research can impact positively on daily work and outcomes (Hicks, 1996). To harness the benefits of a research-active workforce, managers must develop a clear leadership vision and direction. Clear pathways to research training, managerial support, organizational leadership, vision and commitment are vital drivers for encouraging research culture in clinical practice (Atkinson et al., 2008; Lode et al., 2015; O’Byrne & Smith, 2010).

Reported research motivators highlighted respondents’ aspirations for career development, advancing clinical practice, and improving patient and staff experience. Ambition for career progression has been previously identified as a research motivation (Lode et al., 2015); however, funding access and research training availability is essential to support this. Increasing the availability of non-medical research role models and helping service managers to support staff could encourage practice-based research enthusiasts (Melnyk et al., 2004; National Institute for Health and Care Research, 2023a,b; O’Byrne & Smith, 2010). However, for sustainable change, it is essential to develop research strategies, communicate these effectively and place them centrally in organizational policies (Gee & Cooke, 2018).

## Strengths and Limitations

The current study provides new information about mental health non-medical professionals, a group who have rarely been investigated using the current validated study tool. The comparison of results from the current sample with pooled weighted medians and means from all currently available comparable data is also a strength. Limitations include a low response rate, especially among nurses, which may reflect a lack of wider interest among this group of employees in research or may result from questionnaire distribution during the COVID-19 pandemic. However, the response rate was comparable to those reported in a number of other studies (ranked 25/30 among all reported response rates). As a result of under-recruitment there was a margin of error of 8.0% with a confidence level of 95%. Future research should endeavor to maximize response rate. There was no safeguard against multiple participation although, based on feedback received, completing the questionnaire was arduous and deliberate duplication seems unlikely. The primary data were collected in a single NHS Mental Health and Learning Disability Trust and thus results may not be generalizable to other settings. Future research should involve multiple organizations in order to facilitate comparisons and maximize generalizability.

## Implications for Clinical Practice

Research activity in health services is associated with improved patient satisfaction and higher ratings of service quality. However, this survey suggests that non-medical practitioners in mental health services are not adequately prepared for roles in which working with knowledge derived from contemporary clinical research is vital, and where contributing to knowledge generation is at very least desirable. Changing this situation will require a commitment from mental health practitioners to identifying and taking development opportunities that contribute to their individual research capacity. Equally, it requires a commitment from employers to encourage staff and provide opportunities for development. Mental health services should review their organizational approach to growing research capacity and culture in order to maximize the benefits of research activity. There is a need for ongoing educational and developmental interventions to prepare staff in mental health services for roles in research. It makes sense to tailor these interventions for an audience that works in mental health services, and to maximize skills that also transfer to activities such as clinical audit. A joined-up approach across non-medical professions is warranted since there was no evidence of difference in the views of different groups.

## Conclusions

There is ample room for much needed development in research capacity and culture for nurses and allied health professionals working in mental health services. There are indications that the need for development is perhaps even more urgent in this mental health setting, and this will need to incorporate tailored education that includes transformational learning (such as through mentorship) alongside more traditional research training. Organizational infrastructures that improve access to and participation in healthcare research and provide time and resources integrated as part of existing roles. Nursing and allied healthcare professional leaders at a national level should consider development of stronger leadership collaborations between universities and health services to increase the availability and buy in to support clinical academic careers.

## Supplemental Material

sj-docx-1-son-10.1177_23779608241250207 - Supplemental material for Mental Health Nurses’ and Allied Health Professionals’ Individual Research Capacity and Organizational Research Culture: A Comparative Study Supplemental material, sj-docx-1-son-10.1177_23779608241250207 for Mental Health Nurses’ and Allied Health Professionals’ Individual Research Capacity and Organizational Research Culture: A Comparative Study by Geoffrey L. Dickens, Maria Avantaggiato-Quinn, Sara-Jaye Long, Mariyana Schoultz and Nicola Clibbens in SAGE Open Nursing
